# Clinical Implication of PD-L1 Expression in Patients with Endometrial Cancer

**DOI:** 10.3390/biomedicines11102691

**Published:** 2023-10-01

**Authors:** Yeorae Kim, Ala Aiob, Hyojin Kim, Dong Hoon Suh, Kidong Kim, Yong Beom Kim, Jae Hong No

**Affiliations:** 1 Department of Obstetrics and Gynecology, Seoul National University Bundang Hospital, Seongnam 13620, Republic of Korea; yeorae.delight@gmail.com (Y.K.); sdhwcj@naver.com (D.H.S.); kidong.kim.md@gmail.com (K.K.); ybkimlh@snubh.org (Y.B.K.); 2 Department of Obstetrics and Gynecology, Galilee Medical Center, Nahariya 22100, Israel; ala.aiob@gmail.com; 3 Azrieli Faculty of Medicine, Bar Ilan University, Safed 5290002, Israel; 4 Department of Pathology, Seoul National University Bundang Hospital and College of Medicine, Seoul National University, Seongnam 13620, Republic of Korea; hyojinkim7137@snubh.org; 5 Department of Obstetrics and Gynecology, Seoul National University College of Medicine, Seoul 03080, Republic of Korea

**Keywords:** endometrial cancer, PD-L1, clinicopathological, prognosis

## Abstract

This study investigated PD-L1 expression in endometrial cancer, its links with prognostic factors, and survival outcomes in 232 patients. Of these, 73 (31.5%) had PD-L1-positive tumors and 159 (68.5%) had PD-L1-negative tumors. PD-L1 expression significantly correlated with adverse prognostic factors. The PD-L1-positive group had higher rates of high-grade tumors (37.0% vs. 19.1%, *p* = 0.004), deep myometrial invasion (35.6% vs. 24.4%, *p* = 0.004), lymphovascular space invasion (LVSI) (39.7% vs. 25.6%, *p* = 0.023), and lymph node metastasis (7.2% vs. 17.1%, *p* = 0.024) than the PD-L1-negative group. While 5-year progression-free survival (PFS) favored the PD-L1-negative group (94.1% vs. 86.3%), this difference lacked statistical significance (*p* = 0.139). No significant variations emerged in overall survival (OS) (*p* = 0.596) or recurrence rates between the groups. Although outcomes lack statistical significance, they suggest a plausible link between PD-L1 and established adverse prognostic factors, such as histological grade, myometrial invasion depth, LVSI, and lymph node metastasis in endometrial cancer. These insights hint at PD-L1’s potential as an informal prognostic indicator, potentially aiding in endometrial cancer patient management.

## 1. Introduction

Endometrial cancer is one of the most common gynecological cancers in developed countries [1,2]. The incidence of endometrial cancer is estimated to demonstrate a steady annual increase of approximately 1%, which is attributed to an increase in obesity and aging rates [1,2,3,4]. Endometrial cancer generally has a favorable prognosis because of the good surgical outcomes if detected early with symptoms [2,5,6]. The standard treatment for early endometrial cancer is total hysterectomy with bilateral salpingo-oophorectomy (BSO) and pelvic lymph node dissection for surgical staging [1,6]. According to the results of surgical staging, adjuvant radiotherapy with or without platinum-based chemotherapy may be a treatment option depending on high-intermediate risk factors such as age, grade, degree of myometrial invasion, and lymphovascular space invasion (LVSI) [7,8]. However, the survival rate of patients with endometrial cancer has not improved, which is mainly due to the absence of a definitive treatment regimen for patients with advanced or recurrent disease [2,5,9]. Generally, the 5-year relative survival rate for patients with localized or regional endometrial cancer ranges from 69% to 95%, whereas for patients with advanced endometrial cancer with distant metastasis it is only 18% [4,5,10].

Over the past decade, efforts have been made to determine the significant prognostic factors for endometrial cancer and improve the treatment outcomes for patients with the disease [10,11,12]. Immunotherapy is one of the most widely studied emerging approaches to cancer treatment in recent years, especially treatment involving the administration of immune checkpoint inhibitors (ICIs) [5,7]. 

The US Food and Drug Administration (FDA) has approved the administration of ICIs for the treatment of advanced and recurrent endometrial cancer. Among several ICIs, the anti-programmed cell death protein 1 (PD-1) antibody, pembrolizumab, has been approved by the FDA as an efficient and safe alternative for the treatment of advanced or metastatic endometrial cancer [5,13]. In addition, the FDA has approved the assessment of PD-L1 expression as a companion diagnostic test for the use of pembrolizumab in the treatment of cancers other than endometrial cancer. In general, the DNA mismatch repair (MMR) test is used to determine patients with endometrial cancer who are eligible for treatment with PD-1/PD-L1 inhibitors [13].

Even though microsatellite instability (MSI) tests or MMR tests have been used for ensuring endometrial cancer patients meet the criteria for ICIs following the molecular classification, we wondered if these agents could also be used as prognostic factors since PD-1 or PD-L1 inhibitors have been used for treatment. As a matter of fact, PD-L1 is a predictive marker for several cancers, including lung cancer and head and neck cancer [7,14,15]. Although some studies have investigated PD-L1 expression in endometrial cancer, the relationship between PD-L1 and the prognostic factors of endometrial cancer has not yet been clearly defined [7]. Thus, this study aimed to investigate the association between PD-L1 expression and the clinicopathological characteristics of patients with endometrial cancer and to analyze and compare the survival of patients with PD-L1-positive and PD-L1-negative endometrial cancer.

## 2. Materials and Methods

### 2.1. Study Group

This retrospective cohort study was approved by the Institutional Review Board of Seoul National University Bundang Hospital (SNUBH; Seongnam, Republic of Korea; No. B-2207-769-105). The requirement for informed consent was waived owing to the retrospective nature of this study.

Women older than 20 years who were diagnosed with primary endometrial cancer between May 2003 and March 2022 and were treated and followed up at SNUBH were included in this study. The specimens used for diagnosis were stored at SNUBH. Formalin-fixed paraffin-embedded tumor tissues were collected from all the included patients, and every specimen was re-identified by a pathologist (HK). We excluded patients with double primary cancers, sarcoma histology, and insufficient clinicopathological information.

All the included patients were Koreans, and they underwent total hysterectomy, BSO, and pelvic paraaortic lymphadenectomy. Omentectomy was restricted to selected cases. All the tumors were staged according to the International Federation of Gynecology and Obstetrics (FIGO) 2009 criteria, and the clinical and follow-up data of each patient were obtained from clinical records. All included patients were pathologically confirmed to have endometrial cancer. The patients with inoperable advanced-stage disease underwent mass biopsy for pathologic confirmation. The patients who did not show lymph node metastasis, as confirmed through imaging or sentinel lymph node mapping with a frozen biopsy sample, and those who underwent total hysterectomy with BSO without lymph node dissection or sentinel lymph node mapping, because they had stage IA disease, were considered to be without lymph node metastasis. Adjuvant therapy was administered according to the department protocol based on the most recent National Comprehensive Cancer Network (NCCN) guidelines for uterine cancer.

### 2.2. Immunohistochemistry for PD-L1, p53, and MMR Proteins

#### 2.2.1. PD-L1 Expression 

Formalin-fixed, paraffin-embedded tissues were sectioned at a thickness of 4 μm and stained using an automated immunostainer (Ventana Medical Systems, Tucson, AZ, USA) according to the manufacturer’s protocol. The slides were dried at 60 °C for 1 h and deparaffinized at 75 °C for 4 min using EZ Prep (Ventana Medical Systems, Tucson, AZ, USA). The cells were conditioned (heat pre-treatment) at 100 °C for 64 min using a cell conditioning solution that contained Tris/borate/ethylenediaminetetraacetic acid. The anti-PD-L1 22C3 mouse monoclonal primary antibody (Agilent Technologies, Santa Clara, CA, USA) was diluted to 1:50 and applied to the sections, which were then incubated at 37 °C for 32 min. Signals were detected using an Optiview detection kit (Ventana Medical Systems, Tucson, AZ, USA) with streptavidin-biotin staining. Counterstaining was performed for 2 min at room temperature using Mayer’s hematoxylin (ScyTek, Logan, UT, USA). PD-L1 expression was defined if membranous and/or cytoplasmic staining was observed in tumor cells and tumor-associated immune cells. The combined positive score (CPS) was recorded based on the number of PD-L1-positive tumors and immune cells in relation to the total number of tumor cells. PD-L1 positivity was defined as a CPS > 1. 

#### 2.2.2. Microsatellite Instability (MSI) Testing for p53 and MMR Proteins 

Immunohistochemistry analysis was performed using a tissue microarray to evaluate the expression of tumor protein p53 (p53) and two MMR proteins (hMSH6 and PMS2). Staining for p53 was performed using a primary monoclonal antibody (pre-diluted DO-7; Dako, Santa Clara, CA, USA) as previously described [8]. Expression of p53 was considered aberrant if >75% of the cells were strongly positive for p53 (overexpression) or if 0% of the cells were positive (null phenotype). Staining for the MMR proteins was performed using primary monoclonal antibodies against MSH6 (GRBP.P1/2/D4, 1:200; Serotec Inc., Raleigh, NC, USA) and PMS2 (A16-4, 1:200; PharMingen, San Diego, CA, USA). Expression was defined as abnormal if the expression of at least one of the MMR proteins was completely absent from all tumor cell nuclei [16].

### 2.3. Statistical Analyses

To ensure the robustness of our study, we conducted a comprehensive power analysis to determine the appropriate study size. With an anticipated effect size, a desired statistical power of 80%, and an alpha level of 0.05, we determined that a sample size of 232 patients would allow us to effectively detect meaningful differences between the PD-L1-negative and PD-L1-positive groups.

Our analysis encompassed a range of parameters to investigate variations in clinicopathological characteristics. These included age, parity, histology, stage, grade, depth of invasion, LVSI, serum CA125 level, presence of lymph node metastasis based on surgically obtained tissue, adjuvant treatment, and recurrence.

We employed a multifaceted statistical approach to assess distinctions in clinicopathological characteristics between the groups. Continuous variables were compared using the Student *t*-test or the Mann–Whitney U test, depending on the data distribution. Categorical variables, on the other hand, underwent assessment using both Pearson’s Chi-square test and Fisher’s exact test.

Survival outcomes were evaluated using the Kaplan–Meier method, and disparities between the PD-L1-negative and PD-L1-positive groups were analyzed utilizing the log-rank test. Additionally, our analysis extended to multivariate analysis, leading to the formulation of Cox proportional hazard regression models. This facilitated the calculation of adjusted hazard ratios and corresponding 95% confidence intervals. These models allowed us to account for potential confounding variables, thereby providing a deeper understanding of the independent impact of PD-L1 status on survival outcomes.

All statistical analyses were conducted utilizing SPSS Statistics (version 25.0; IBM Corp., Armonk, NY, USA). Statistical significance was established at a *p*-value threshold of less than 0.05. Collaboration with the SNUBH statistics team strengthened our dedication to upholding data integrity. Furthermore, our findings were meticulously validated through a thorough data verification process and rigorous consultations

## 3. Results

We identified a cohort of 285 Korean women who were diagnosed with histologically confirmed endometrial cancer and underwent follow-up at SNUBH from May 2003 to March 2022. A comprehensive examination of the medical records was conducted for each screened patient within this singular medical center. Through this comprehensive chart review process, we excluded individuals with double-primary cancer, those lacking PD-L1 test results, or those exhibiting insufficient medical or pathological information. Ultimately, the study encompassed a total of 232 patients. The demographic and clinicopathological characteristics of these 232 patients with endometrial cancer are presented in Table 1. The median age of the patients was 56.8 (25–91) years. A total of 202 (87.1%) patients had endometrioid histologic type, whereas 30 (12.9%) patients had cancers of other histological types such as serous, clear cell carcinoma, mixed, and neuroendocrine carcinoma. A total of four patients were diagnosed with mixed-type endometrial cancers, and in all cases, the mixed-type tissue was comprised of both serous and endometrioid components. Most of the patients had stage 1 disease (79.3%, 184/232), low-grade endometrial cancer (74.9%, 173/232), and no LVSI (69.9%, 160/232). 

Commencing with the presentation of clinical data, it is noteworthy that within the studied cohort, sentinel lymph node mapping was carried out in ninety-four patients, while pelvic lymph node dissection was performed in seventy-seven patients. The findings of the procedures showed that two women had pelvic lymph node metastasis. 

Adjuvant treatment after surgery was administered to 94 patients, 51 of whom received radiation therapy. Of the 51 patients who received radiation therapy, eight received radiotherapy with chemotherapy, three received radiotherapy with chemotherapy administered in a sandwich regimen, two received brachytherapy with chemotherapy, two received palliative radiotherapy following chemotherapy, and the remaining patients received radiotherapy following adjuvant chemotherapy. A total of 33 patients received chemotherapy only. Of these 33 patients, two had stage IVB disease and did not undergo surgery, one patient received nine cycles of paclitaxel-carboplatin, and the others received seven cycles of paclitaxel-carboplatin with palliative radiotherapy administered to the pelvic cavity. Twelve patients received chemoradiotherapy adapted to the PORTEC3 trial regimen [17], whereas three patients received chemotherapy with vaginal brachytherapy. 

According to the immunohistochemistry analysis of PD-L1 expression, all the included patients were categorized into the PD-L1-positive group, which was the study group (68.5%, 159/232), and the PD-L1-negative group, which was the control group (31.5%, 73/232). 

As outlined in Table 1, it is evident that the PD-L1-positive group exhibited a higher incidence of patients with high-grade endometrial cancer compared to the PD-L1-negative group (37% vs. 19.1%, *p* = 0.004). Similarly, a greater proportion of patients in the PD-L1-positive group presented with characteristics such as deep invasion (35.6% vs. 24.4%, *p* = 0.004), LVSI (39.7% vs. 25.6%, *p* = 0.023), and lymph node metastasis (17.1% vs. 7.2%, *p* = 0.024).

Furthermore, notable disparities were observed between the two groups in terms of invasion depth, with patients in the PD-L1-positive group displaying significantly deeper lesions compared to those in the PD-L1-negative group (7.88 ± 10.68 vs. 5.71 ± 8.64, *p* = 0.012), as assessed by the researched depth of invasion (mm). However, it is noteworthy that no statistically significant differences were found between the two groups concerning age, parity, histological type, CA125 level, FIGO stage, recurrence, and mortality.

For the MSI test, the proportion of MSI-high results in the PD-L1-positive group was higher than that in the PD-L1-negative group (85.7% vs. 44.0%, *p* = 0.005). Deficiency of MSH6 and PMS2 was more common in the study group than in the control group (9.7% vs. 4.5%, *p* = 0.006; 31% vs. 12.3%, *p* = 0.002, respectively). However, there were no statistically significant differences in the expression of p53, estrogen or progesterone receptors, and HER2/neu between the two groups (Table 2).

**Figure 1:**
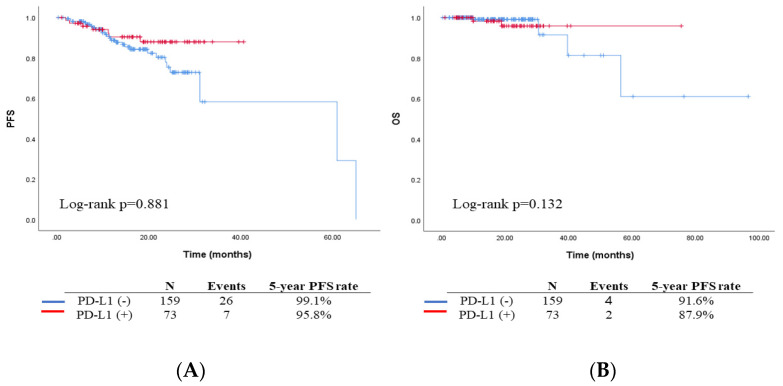
Survival outcomes with Kaplan-Meier curves according to the PD-L1 expression (red line: positive vs. blue line: negative); (**A**) 5-year PFS of the total study population (99% vs. 96%, *p* = 0.881); (**B**) 5-year OS of the total study population (92% vs. 88%, *p* = 0.132).

With respect to the patient’s survival outcomes, it is noteworthy that the median follow-up duration encompassed 17.2 months (range: 0.3–92.5 months). Within this timeframe, thirty-three cases (13.4%) experienced recurrences, and five cases (2.2%) resulted in mortality. Upon conducting survival analysis, no statistically significant distinctions were observed in terms of progression-free survival (PFS) or overall survival (OS) (*p* = 0.596) between the two groups, as illustrated in Figure 1. It is, however, important to note that the PD-L1-negative group exhibited a comparatively improved PFS compared to the PD-L1-positive group (5-year PFS: 94.1% vs. 86.3%, *p* = 0.139).

## 4. Discussion

Identifying biomarkers that can predict patients with endometrial cancer who are most likely to respond to immunotherapy is essential. In contrast to other common cancers, the incidence and annual mortality rates of endometrial cancer are increasing [18,19]. Only a few therapeutic alternatives have been available for women with advanced or recurrent endometrial cancer in recent years [20]. Immune-based therapy could have a significant therapeutic effect in selective patients with endometrial cancer. In the present study, we evaluated the association between PD-L1 expression and the clinicopathological factors of endometrial cancer. In addition, we analyzed and compared survival and recurrence in patients with PD-L1-positive and PD-L1-negative endometrial cancer. 

In the assessment of PD-L1 immunohistochemistry outcomes, we elected to employ the Combined Positive Score (CPS) methodology, as opposed to employing the Type-specific Scoring (TPS) or Individual Cell Scoring (ICS) approaches. It is imperative to acknowledge that the evaluation of PD-L1 immunohistochemistry results may be influenced by various factors, including divergent assay standardization protocols and distinct scoring systems utilized to gauge PD-L1 positivity. Within the specific context of endometrial cancer investigation, our analysis suggests notable methodological advantages associated with CPS, particularly when juxtaposed with cell type-specific scoring methods such as TPS and ICS [21].

Since 2018, SNUBH has implemented a systematic Lynch syndrome screening protocol for all patients diagnosed with endometrial cancer. Subsequent to the acquisition of surgical tissue biopsies, our institutional practice mandates the comprehensive analysis of MSH6, PMS2, PD-L1, and p53 as part of the standard diagnostic assessment conducted by the pathology department. Consequently, the present study concurrently evaluates MSH6, PMS2, and PD-L1 markers. The implementation of this particular endometrial cancer screening methodology was a result of careful deliberation, with considerations encompassing cost-effectiveness and the intricacies of the Korean health insurance system. However, it is noteworthy that the inclusion of ER/PR assessment was not uniformly administered to all patients, consistent with the aforementioned strategic approach. In this context, if MSH6 and PMS2 were proficient, then we interpreted them as proficient MMR proteins (Table 2) [22,23,24]. When the two MMR minor proteins (hMSH6 and PMS2) were proficient, PD-L1 tended to be positive (*p* = 0.006/0.002). In addition, the rate of lymph node metastasis was significantly higher in the PD-L1-positive group than in the PD-L1-negative group (17.1% vs. 7.2%, *p* = 0.024). This finding is consistent with the results of a previous study [25].

In the context of our present study, the cumulative percentage of patients diagnosed with endometrial cancer and demonstrating positive PD-L1 expression amounted to 31.5%. This observation harmonizes with results from earlier inquiries, where previously reported rates of PD-L1 expression also exhibited a similar trend [7,26]. It has been established that PD-L1 expression could predict better response rates to PD-1/PD-L1 inhibition therapy in patients with various types of cancer [27]. Additionally, positive PD-L1 expression has been shown to be a poor prognostic predictor for some solid tumors such as breast cancer and is a favorable prognostic indicator for others [28,29]. Findings on the predictive value of PD-L1 for endometrial cancer are debatable [30,31,32,33]. 

The results of the present study showed that there was no significant difference in histological type (endometroid or non-endometroid) between PD-L1-positive and PD-L1-negative tumors. This finding is consistent with the results of a previous prospective study, which indicated that PD-L1 expression is not associated with tumor histological type [8]. However, other studies have shown that PD-L1 expression is more common in endometrioid carcinomas than in tumors with non-endometrioid histology [34,35]. However, another study indicated that the only significant difference between endometroid and non-endometroid tumors is PD-L1 positivity in immune cells and not in tumor cells [36].

Upon juxtaposing the outcomes of this study with those of previous investigations, it becomes evident that the incidence of PD-L1 positivity exhibited a distinct elevation in high-grade endometrial carcinomas, as compared to their low-grade counterparts. This alignment of findings reinforces the conclusions drawn from prior scholarly inquiries [33]. Moreover, the prevalence of PD-L1 positivity manifested a higher frequency within cases characterized by LVSI and myometrial invasion, in contrast to instances lacking these factors. This finding harmonizes with observations previously documented in the literature [30]. Collectively, these findings imply a plausible link between the microcystic elongated fragmented pattern of endometrial cancer invasion and the expression of PD-L1. This association can be attributed to the morphological connections with metaplastic changes and LVSI [37]. However, it is important to note that some studies have not unveiled any significant correlations between PD-L1 expression and various clinicopathological parameters of endometrial cancer [31,35]. 

The Kaplan–Meier survival curve showed a crossover; thus, we could not observe a significant difference in survival rates according to PD-L1 expression (Figure 1). The subgroup analysis according to tumor grade, disease stage, and lymph node metastasis showed that PD-L1 expression was not a significant prognostic factor for endometrial cancer (Supplemental Tables S1–S3). Further assessment of the reason for the crossover in the survival analysis result indicated that the effect of PD-L1 as a predictive marker was slightly weaker in the lower tumor grade group than in the high tumor grade group. We assumed that the experimental results may change if the follow-up period is extended. Although the survival analysis showed no significant difference in survival between the two groups, PD-L1 was associated with known poor prognostic factors such as deep myometrial invasion, high tumor grade, and lymph node metastasis. 

The strength of this study is that it was conducted using a large number of cases selected from a single tertiary medical center. However, the study was limited by its retrospective cohort design. Another limitation is that several factors that could interfere with the disease course were not estimated. In addition, the follow-up period in some cases was short, thereby limiting the value of PFS and OS data in these cases. The analysis of PD-L1 expression has been performed for every case of endometrial cancer in our hospital since 2018 as part of the screening for Lynch syndrome and screening for candidates for ICI therapy. 

Unfortunately, analysis of PD-L1 expression was not conducted in all cases of endometrial cancer before 2018. In addition, PD-L1 expression was not analyzed in cases of endometrial cancer before late 2010. In addition, we evaluated PD-L1 levels in tumor cells, not in immune cells. Given that our study was not originally designed to assess immune cells, the dataset lacks information pertaining to PD-L1 expression in these cells [26]. Therefore, should the opportunity arise for further research, a more comprehensive analysis of PD-L1 will be conducted to elucidate these aspects in greater detail. Although we enrolled all patients with endometrial cancer who met the diagnosis criteria, the present study has a limitation because this is a retrospective study with a medical chart in a single medical center. In addition, there have been various suggestions about the scoring system of PD-L1. Thus, it is advisable to interpret the results of the experiments performed with the PD-L1 with caution and to plan future experiments with these results in mind. 

## 5. Conclusions

This study showed that there was no statistically significant difference in PFS and OS between patients with PD-L1-positive endometrial cancer and those with PD-L1-negative endometrial cancer. Nevertheless, PD-L1 expression was significantly correlated with poor prognostic factors of endometrial cancer, such as histological grade, myometrial invasion, LVSI, and lymph node metastasis.

## Figures and Tables

**Table 1:** Clinicopathological characteristics in patients with PD-L1 positive and negative expression.

Parameters	No (%)			
	Total	PD-L1 Negative (159)	PD-L1 Positive (73)	*p*-Value
Age	56.8 (25.7–91.4)	56.6 ± 10.9	57.0 ± 10.6	
Parity	1.6 (0–6)			
0	49 (21.1)	36 (22.6)	13 (17.8)	0.489
≥1	183 (78.9)	123 (77.4)	60 (82.2)	
Histology				
Endometrioid	202 (87.1)	137 (86.2)	65 (89.0)	0.353
Others	30 (12.9)	22 (13.8)	8 (11.0)	
FIGO stage				0.064
I	184 (79.3)	131 (82.4)	53 (72.6)	
II, III, IV	48 (20.7)	28 (17.6)	20 (27.4)	
**FIGO grade**				**0.004**
Low	173 (74.9)	127 (80.4)	46 (63.0)	
High	58 (25.1)	31 (19.6)	27 (37.0)	
**Depth of invasion**				**0.004**
EM only	71 (31.0)	59 (37.8)	12 (16.4)	
<1/2	94 (41.0)	59 (37.8)	35 (47.9)	
≥1/2	64 (27.9)	38 (24.4)	26 (35.6)	
**LVSI**				
No	160 (69.9)	116 (74.4)	44 (60.3)	**0.023**
Yes	69 (30.1)	40 (25.6)	29 (39.7)	
CA125 elevation				0.557
<35	168 (74.0)	114 (74.0)	54 (74.0)	
≥35	59 (26.0)	40 (26.0)	19 (26.0)	
**Lymph node metastasis**				**0.024**
No	200 (89.7)	142 (92.8)	58 (82.9)	
Yes	23 (10.3)	11 (7.2)	12 (17.1)	
Adjuvant treatment				0.200
No	138 (59.5)	98 (61.6)	40 (54.8)	
Yes	94 (40.5)	61 (38.4)	33 (45.2)	
Recurrence				0.120
No	199 (85.8)	133 (83.6)	66 (90.4)	
Yes	33 (14.2)	26 (16.4)	7 (9.6)	
Death				0.614
No	226 (97.4)	155 (97.5)	71 (97.3)	
Yes	6 (2.6)	4 (2.5)	2 (2.7)	
Follow-up period, month	17.2 (0.39–96.56)			
**Depth of invasion (average ± SD, mm)**		5.71 ± 8.64	7.88 ± 10.68	**0.012**

Abbreviations: FIGO, International Federation of Gynecology and Obstetrics; CA125, cancer antigen 125; EM, endometrium; LVSI, lymphovascular space invasion; RM, resection margin; PM, parametrium; CPS, combined positive score.

**Table 2:** Comparison of immunohistochemical staining results between PD-L1 positive and negative groups (Chi-square). The **bold** represents significant values, with a *p*-value less than 0.05.

	PD-L1 Status		*p*-Value
	Negative (%)	Positive (%)	
P53			0.551
Wild type	128 (87.7)	60 (88.2)	
Mutant type	18 (12.3)	8 (11.8)	
**MSI test (n = 46)**			**0.005**
MSS or MSI-L	14 (56.0)	3 (14.3)	
MSI-H	11 (44.0)	18 (85.7)	
MMR proteins			
**hMSH6**			**0.006**
Proficient	147 (93.6)	58 (80.6)	
Deficient	7 (4.5)	7 (9.7)	
**PMS2**			**0.002**
Proficient	133 (86.4)	49 (69.0)	
Deficient	19 (12.3)	22 (31.0)	
Estrogen receptor (n = 73)			0.917
Negative	11 (22.9)	6 (24.0)	
Positive	37 (77.1)	19 (76.0)	
Progesterone receptor (n = 74)			0.675
Negative	14 (28.6)	6 (24.0)	
Positive	35 (71.4)	19 (76.0)	
HER2/neu (n = 22)			0.309
Negative	12 (70.6)	2 (40.0)	
Positive	5 (29.4)	3 (60.0)	

Abbreviations: PD-L1, Programmed death-ligand 1; MSI, microsatellite instability; MSS, microsatellite stable; MSI-L, microsatellite instability-low; MSI-H, microsatellite instability-high; HER2/neu, human epidermal growth factor receptor-2.

## Data Availability

The datasets used and/or analyzed during the current study are available from the corresponding author upon reasonable request.

## Supplementary Materials

The following supporting information can be downloaded at: https://www.mdpi.com/article/10.3390/biomedicines11102691/s1. Table S1: Progression-free survival (univariate analysis); Table S2. Multivariate analysis of progression-free survival; Table S3. Subgroup analysis for adjuvant treatment group: progression-free survival (univariate analysis).
