# Supplementary material for: Clinical Implication of PD-L1 Expression in Patients with Endometrial Cancer
Source: Biomedicines. 2023 Oct 1;11(10):2691. doi: 10.3390/biomedicines11102691 (PMC10603999; doi:10.3390/biomedicines11102691)
Supplement: Supplementary file 1 [file biomedicines-11-02691-s001.zip › biomedicines-2569266-supplementary.pdf]

Table S1. Univariate analysis of PFS

Parameters	aHR	95% CI	p-value
PD-L1	0.528	0.227–1.230	0.139
**Invasion > 1/2**	6.927	3.125–15.354	<0.001
**Grade 3**	6.143	2.882–13.093	<0.001
**Stage II and above**	7.385	3.529–15.456	<0.001
**Lymph node metastasis**	6.394	2.870–14.244	<0.001
**Other histology**	4.207	2.034–8.700	<0.001
**Serum CA125 ≥ 35**	3.973	1.936–8.154	<0.001
MSI-H	0.641	0.263–1.563	0.328
**p53 mutant type**	4.969	2.020–12.226	<0.001

Abbreviations: aHR, adjusted hazard ratio; CI, confidence interval

Table S2. Multivariate analysis of PFS

Parameters	aHR	95% CI	p-value
PD-L1	0.404	0.140–1.164	0.093
**Invasion > 1/2**	4.419	1.596–12.237	0.004
**Grade 3**	3.645	1.253–10.601	0.018
**Stage II and above**	4.990	1.741–14.303	0.003
Lymph node metastasis	0.482	0.106–2.195	0.346
Other histology	1.611	0.367–7.078	0.528
Serum CA125 ≥ 35	0.382	0.083–1.753	0.216
MSI-H	0.426	0.119–1.525	0.190
P53 mutant type	0.641	0.146–2.817	0.556

Abbreviations: aHR, adjusted hazard ratio; CI, confidence interval

Table S3. Subgroup analysis for adjuvant treatment group: progression-free survival (univariate analysis)

Parameters	Univariate			Mutivariate	
	aHR	95% CI	p-Value	aHR	p-value
PD-L1	0.442	0.186–1.053	0.065		
Invasion > 1/2	0.903	0.385–2.119	0.814		
Grade 3	0.951	0.404–2.241	0.909		
Stage II and above	0.879	0.357–2.164	0.778		
Tumor size over 2 cm	0.698	0.316–1.542	0.374		
Lymph node meta	0.530	0.235–1.195	0.126		
Other histology	1.318	0.583–2.978	0.508		
Serum CA125 ≥ 25	0.945	0.439–2.036	0.885		
**MSI-H**	**0.188**	**0.040–0.886**	**0.035**	0.226	0.303.
**P53 mutant type**	**2.684**	**1.034–6.973**	**0.043**	0.000	0.995

Abbreviations: aHR, adjusted hazard ratio; CI, confidence interval
